# Paclitaxel-resistant advanced recurrent breast cancer: a case of partial response due to addition of bevacizumab to paclitaxel therapy: a case report

**DOI:** 10.1186/1756-0500-6-254

**Published:** 2013-07-06

**Authors:** Kazuo Ishizuna, Jun Ninomiya, Makoto Kojima, Miho Kawashima, Miwako Nozaki, Hidetsugu Yamagishi, Yoshihiko Ueda, Masatoshi Oya

**Affiliations:** 1 Breast Center, Dokkyo Medical University Koshigaya Hospital, 2-1-50 Minami-Koshigaya, Saitama 343–8555, Koshigaya, Japan; 2 Ninomiya Hospital, 491–6 Shin-eicho, Soka, Saitama 340–0056, Koshigaya, Japan; 3 Department of Radiology, Dokkyo Medical University Koshigaya Hospital, 2-1-50 Minami-Koshigaya, Saitama 343–8555, Koshigaya, Japan; 4 Department of Pathology, Dokkyo Medical University Koshigaya Hospital, 2-1-50 Minami-Koshigaya, Saitama 343–8555, Koshigaya, Japan

**Keywords:** Breast cancer, Bevacizumab, Paclitaxel

## Abstract

**Background:**

Paclitaxel plus bevacizumab have shown a high response rate and prolonged progression-free survival in metastatic breast cancer patients. However, overall survival was not prolonged. Thus, no conclusion has been made on the effectiveness of bevacizumab. In our report, taxane plus bevacizumab were used to treat a metastatic breast cancer patient with taxane resistance, and a good therapeutic result was obtained.

**Case presentation:**

The patient was a 68-year-old woman with a non-contributory history. In September 2004, she underwent a pectoral muscle-conserving mastectomy with axillary dissection for right-sided breast cancer (pT3N0M0-stage IIB, estrogen receptor positive, progesterone receptor negative, and human epidermal growth factor receptor type 2 negative). Adjuvant therapy consisted of 6 cycles of cyclophosphamide, epirubicin and fluorouracil, and subsequent oral anastrozole. In August 2007, the patient developed a recurrence in the left axillary lymph node. The chemotherapy was changed to high-dose toremifene, and radiation therapy was also performed. The patient achieved a complete response. In April 2009, CT showed left axillary lymph node enlargement once again and multiple lung metastases. Hormone therapy was changed to exemestane and long-term stable disease was achieved. In March 2011, the lung and left axillary lymph node metastases were enlarged and progressive disease was noted. Thus, the tumors were determined to be resistant to hormone therapy, and weekly paclitaxel was begun in May. Since partial response was achieved, this therapy was continued. In December, CT showed that lung and axillary lymph node metastases were enlarged and progressive disease was observed. Therefore, the tumors were determined to be resistant to paclitaxel. In January 2012, bevacizumab and weekly paclitaxel were begun. In April, lung and axillary lymph node metastases were reduced in size, and partial response was achieved. Thereafter the same treatment has been continued, and the patient has been followed up without clinical exacerbation as of January 2013.

**Conclusion:**

Taxane plus bevacizumab were used to treat a metastatic breast cancer patient with taxane resistance, and a good therapeutic result was obtained. This result is considered important in increasing treatment options for patients with taxane resistance or patients using adjuvant taxane-based therapy and in examining the effectiveness of bevacizumab in metastatic breast cancer patients.

## Background

It is often difficult to cure metastatic and recurrent breast cancer, except for some local recurrences. Improvement of QOL and extension of survival are the objectives of treatment for metastatic and recurrent breast cancer. In recent years, various new drugs have been used clinically in an effort to achieve these objectives.

Bevacizumab is a humanized monoclonal antibody that targets vascular endothelial growth factor (VEGF), which is a major regulator of angiogenesis. In Japan, its indications are colon cancer and lung cancer and have expanded to include breast cancer in September 2011.

In this report, we describe a case of paclitaxel (PTX) resistant advanced recurrent breast cancer that achieved partial response due to addition of bevacizumab to paclitaxel therapy. We also include a brief literature review.

## Case presentation

**Figure 1:**
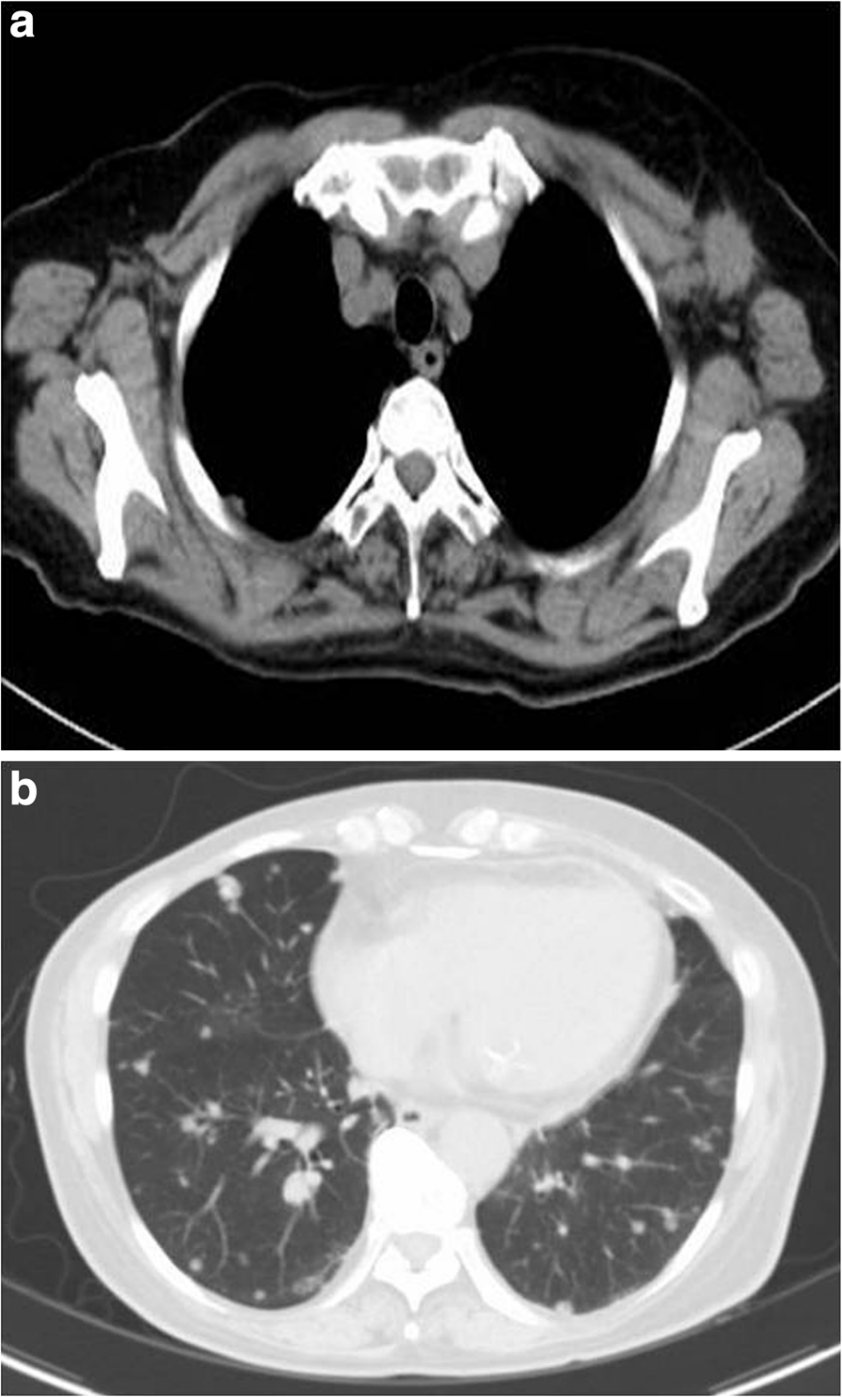
**a and b (CT from December 2011).** CT showed a left axillary lymph node that had enlarged to 2.5 cm and lung metastases that had also enlarged.

The patient was a 68-year-old postmenopausal woman with a non-contributory history. In September 2004, she underwent a pectoral muscle-conserving mastectomy with axillary dissection for right-sided breast cancer. Pathological diagnosis was papillotubular carcinoma, n = 0/12, nuclear grade 1, ly+, v-, estrogen receptor positive, progesterone receptor negative, and human epidermal growth factor receptor type 2 negative (UICC classification: pT3N0M0-stage IIB). Adjuvant therapy consisted of 6 cycles of CEF (cyclophosphamide 75 mg/m^2^ (days 1–14), epirubicin 60 mg/m^2^ (days 1 and 8), and fluorouracil 500 mg/m^2^ (days 1 and 8), every 4 weeks) and subsequent oral anastrozole (1 mg/day). In August 2007, the patient developed a recurrence in the left axillary lymph node. The chemotherapy was changed to high-dose toremifene (120 mg/day), and radiation therapy was also performed (left axilla: 63 Gy). The patient achieved a complete response (CR) in March 2008. In April 2009, CT showed left axillary lymph node enlargement once again and multiple lung metastases. Hormone therapy was changed to exemestane (25 mg/day) and long-term stable disease was achieved. In March 2011, the lung and left axillary lymph node metastases were enlarged and progressive disease was noted. Thus, the tumors were determined to be resistant to hormone therapy. In May, weekly paclitaxel was begun (80 mg/m^2^, 3 weeks on and 1 week off). Since partial response was achieved, this therapy was continued. In December 2011, CT showed that lung and axillary lymph node metastases were enlarged and progressive disease was observed. Therefore, the tumors were determined to be resistant to PTX (tumor markers, CEA: 2.8 ng/ml, CA 15–3: 27.7 U/ml) (Figure 1a and b). Since the patient became resistant to PTX and developed progressive disease, a change to other drugs was considered. However, metastases enlarged only slightly. Thus, PTX was continued in combination with bevacizumab whose indications had been newly expanded to include breast cancer. PTX was continued to exhaust the therapeutic options based on taxanes – a key drug for metastatic and recurrent breast cancer.

**Figure 2:**
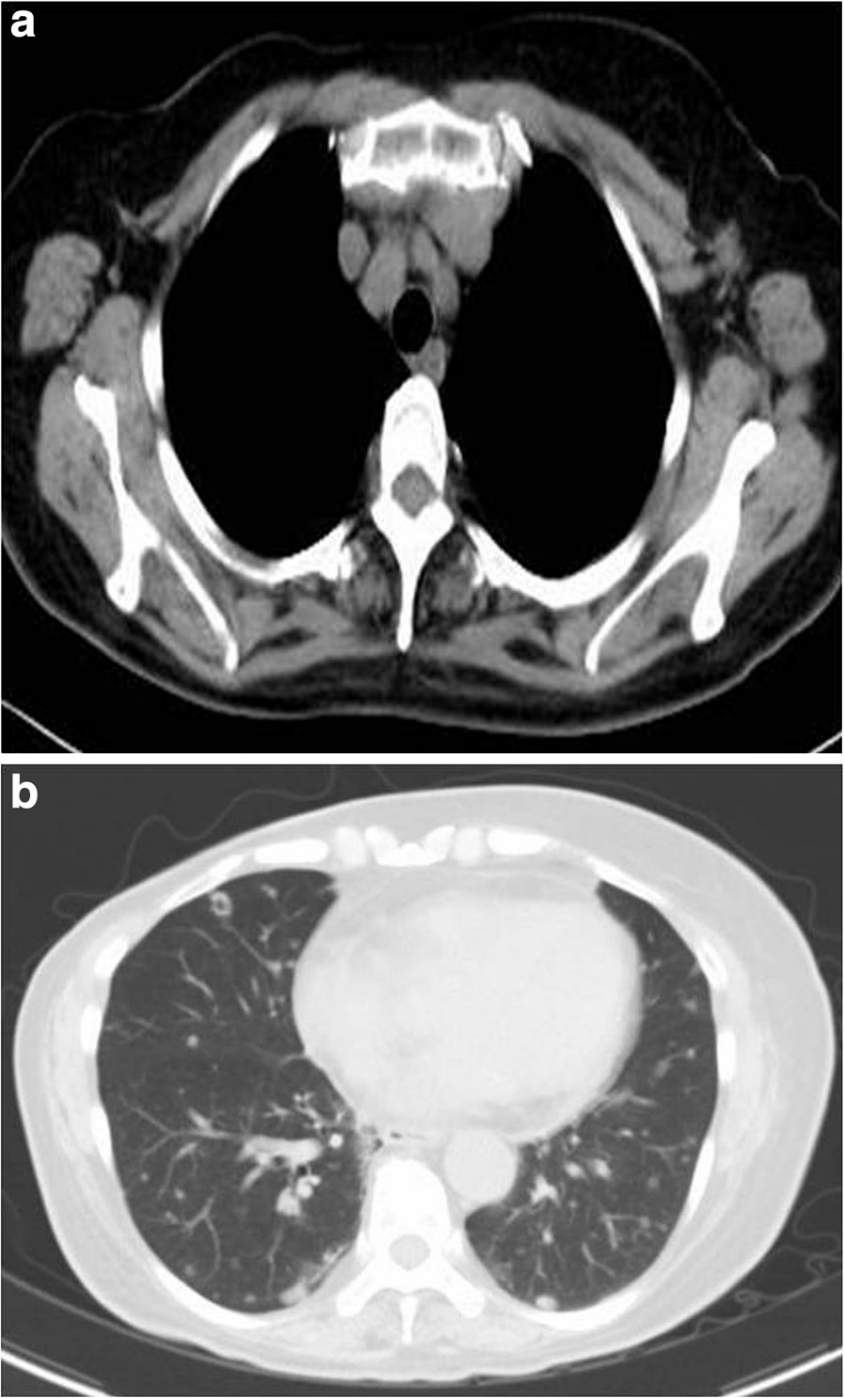
**a and b (CT from April 2012).** Three months after bevacizumab and weekly PTX were begun, the left axillary lymph node reduced in size to 1.4 cm and the lung metastases also became smaller.

In January 2012, bevacizumab and weekly PTX were begun (paclitaxel 90 mg/m^2^, 3 weeks on and 1 week off; bevacizumab 10 mg/kg, once every 2 weeks). In April, CT showed reduction of size of lung and axillary lymph node metastases, and partial response was achieved (tumor markers, CEA: 2.0 ng/ml, CA 15–3: 20.9 U/ml) (Figure 2a and b). Thereafter the same treatment has been continued, and the patient has been followed up without clinical exacerbation as of January 2013.

## Discussion

Tumor growth requires oxygen and nutrients. Tumors vessels are needed to supply sufficient oxygen and nutrients as the tumor size increases. Therefore, angiogenesis plays an important role in tumor growth and metastasis. VEGF is a major regulator involved in tumor angiogenesis, growth, and metastasis. It acts as a ligand and binds to VEGF receptors on the endothelial cell surface [1,2].

The molecularly targeted drug bevacizumab (Avastin; Genentech, South San Francisco, CA) selectively binds to a VEGF family member, VEGF-A. Bevacizumab inhibits binding of VEGF-A to VEGF receptors (VEGFR-1, VEGFR-2, and neuropilin 1) expressed on endothelial cells, thereby blocking the signal transduction pathway. It inhibits angiogenesis in tumor tissue and suppresses tumor growth as a result [3]. In addition, it has been shown to normalize the vascular structure, decrease vascular permeability, and lower increased tumor interstitial pressure [4,5]. When the tumor interstitial pressure is reduced by bevacizumab, paclitaxel delivery to the tumor tissue is improved. Thus, the drug concentration increases in the tumor tissue [6].

Our patient developed resistance to PTX and subsequently achieved a partial response when bevacizumab was added to PTX therapy. The reason is that bevacizumab inhibits angiogenesis and improves paclitaxel delivery into tumor tissue.

In 2007, there was a report on a phase III randomized trial (E2100) which compared paclitaxel plus bevacizumab and paclitaxel alone as initial chemotherapy for patients with untreated advanced and recurrent breast cancer (n = 722). Although the overall survival was not significantly prolonged (median: 26.7 months vs 25.2 months, respectively, HR: 0.88, p = 0.16), the median progression-free survival was prolonged (11.8 months vs 5.9 months, HR: 0.60, p < 0.001) and the response rate increased (36.9% vs 21.2%, p < 0.001) [7]. In the U.S., bevacizumab was promptly approved for metastatic breast cancer in February 2008 based on these results. In Japan, its indications were expanded to include breast cancer patients in September 2011.

When additional trials were conducted (AVADO and RIBBON-1 trials), the results showed that the progression-free survival was prolonged and the response rate increased. However, the overall survival was not prolonged. In addition, the results suggested that the risks for adverse events could outweigh the benefits [7-9]. In November 2011, the FDA revoked the approval of bevacizumab for breast cancer. At the present time, it is necessary to thoroughly examine the risks and benefits of bevacizumab.

When bevacizumab is used in combination with another chemotherapeutic agent, a high response rate can be obtained. Thus, bevacizumab is currently considered a drug with major benefits, particularly in patients with life-threatening metastasis. When patients develop resistance to taxane during adjuvant therapy (as in our patient), our results suggest that the combination therapy of taxane and bevacizumab can be considered for treatment. Thus, our finding can have major significance. In the E2100 trial, metastatic breast cancer patients who had a history of adjuvant taxane chemotherapy were examined. Paclitaxel plus bevacizumab significantly prolonged progression-free survival as compared with paclitaxel alone (12 months vs 3 months, respectively; HR: 0.46, P < 0.001) [7].

In Japan, it has not been very long since bevacizumab has been indicated for metastatic and recurrent breast cancer. However, since it has not been shown to prolong survival, its blind use is also not recommended from the aspect of health economics [10]. However, bevacizumab has been shown to achieve a high response rate and to prolong progression-free survival. Thus, it could be very beneficial depending on the case. In the future, more cases should be accumulated, and it would be necessary to identify a subset of patients in which bevacizumab will be more effective.

## Conclusion

We reported a case of paclitaxel-resistant metastatic recurrent breast cancer that achieved partial response due to addition of bevacizumab to paclitaxel therapy. The clinical value of bevacizumab has not yet been established in treating advanced and recurrent cancer. It will be necessary to accumulate more cases.

## Consent

Written informed consent was obtained from the patient for publication of this manuscript and any accompanying images. A copy of the written consent is available for review by the Editor-in-Chief of this journal.

## Abbreviations

VEGF: Vascular endothelial growth factor; PTX: Paclitaxel.

## Competing interests

The authors declare that they have no competing interests.

## Authors’ contributions

KI, JN, MK, MK and MN participated in the clinical diagnosis. HY and YU performed the histological examination. KI and JN contributed to drafting of the manuscript. MO supervised the concept and design of the manuscript. All authors read and approved the final manuscript.
